# Time and age trends in morning and evening protein intakes of German children and adolescents

**DOI:** 10.1017/jns.2018.1

**Published:** 2018-03-22

**Authors:** Sarah Roßbach, Tanja Diederichs, Christian Herder, Anette E. Buyken, Ute Alexy

**Affiliations:** 1 IEL-Nutritional Epidemiology, DONALD Study, University of Bonn, Heinstueck 11, Dortmund 44225, Germany; 2 Institute of Nutrition, Consumption and Health, Faculty of Natural Sciences, University Paderborn, Warburger Strasse 100, Paderborn D-33098, Germany; 3 Institute for Clinical Diabetology, German Diabetes Center, Leibniz Center for Diabetes Research at Heinrich Heine University Düsseldorf, Auf'm Hennekamp 65, Düsseldorf 40225, Germany; 4 German Center for Diabetes Research (DZD), Ingolstädter Landstrasse 1, München-Neuherberg 85764, Germany

**Keywords:** Morning protein intake, Evening protein intake, Time trends, Age trends, Children, DONALD, Dortmund Nutritional and Anthropometric Longitudinally Designed, E%, percentage of energy intake

## Abstract

The present study describes time and age trends in morning and evening protein intakes and sources among German children and adolescents from 1985 to 2014. A total of 9757 three-day weighed dietary records of 1246 3- to 18-year-old participants of the Dortmund Nutritional and Anthropometric Longitudinally Designed (DONALD) study were analysed using polynomial mixed-effects regression models. Morning protein intake increased over the study period by approximately 1 % of morning energy intake (linear trend *P* < 0·0001), with the youngest and the oldest children having the highest protein intake (linear, quadratic trend *P* < 0·0001). Evening protein intake increased over time by approximately 2 % of evening energy intake in girls (linear trend *P* < 0·0001) and 1 % of evening energy intake in boys (quadratic trend *P* = 0·0313), with decreasing intake with age (girls: linear trend *P* < 0·0001; boys: linear trend *P* = 0·0963). Time trends were largely due to increases in protein from ‘starchy foods’. In conclusion, morning and evening protein intakes increased modestly between 1985 and 2014; these increases were, however, not accompanied by increases in traditional protein sources (i.e. meat or dairy products).

While carbohydrates and fat have been the focus of the discussion on the obesity epidemic, protein has been largely disregarded^(^^1^^)^. According to the ‘protein leverage hypothesis’ a fixed protein target evokes greater increases in fat and carbohydrate intakes if the ratio of protein to carbohydrates and fat within the diet is low^(^^1^^)^. On the other hand, the ‘early protein hypothesis’ postulates that a high protein consumption early in life in excess of metabolic requirements predisposes to an increased risk for obesity in later life^(^^2^^)^. Also due to the fact that protein is the most satiating macronutrient^(^^3^^)^, protein intake should receive more attention. However, to date the most favourable macronutrient distribution for health remains controversial^(^^4^^)^.

In recent years, increasing evidence further suggests a role of timing of food intake for metabolic health. The alignment of food intake with metabolic processes that follow circadian rhythms is one potential explanation. Particularly morning and evening intakes are discussed as relevant in this context. Initial studies also show a potential relevance of morning and evening protein intake for satiety and metabolism: For instance, a protein-rich breakfast might reduce appetite and subsequent-meal food intake^(^^5^^)^ and increase energy expenditure and fat oxidation in contrast to a carbohydrate-based breakfast^(^^6^^)^. Protein consumed at breakfast has also been associated with enhanced satiety compared with protein intake at other meals^(^^7^^)^. Regarding evening intake, popular weight-loss diets advocate a preference of high protein intake, specifically in evening meals^(^^8^^,^^9^^)^. Preliminary evidence further suggests that morning and evening macronutrient intakes during childhood and adolescence are of relevance for the development of body composition and risk markers of type 2 diabetes in young adulthood^(^^10^^,^^11^^)^.

While total daily protein intake remained fairly stable in healthy German children^(^^12^^,^^13^^)^, data on time trends in daytime-specific macronutrient intakes are lacking. Therefore, we investigated time (1985–2014) and age (3–18 years) trends in morning and evening protein intakes of German children and adolescents using daytime-specific nutritional intake data from 3-d weighed dietary records from the Dortmund Nutritional and Anthropometric Longitudinally Designed (DONALD) study by means of polynomial mixed-effects regression models. Major food groups contributing to morning and evening protein intakes were also investigated.

## Subjects and methods

### Study population

The DONALD study is an ongoing open cohort study which collects information on diet, growth and metabolism of healthy participants between infancy and adulthood since 1985. The DONALD study is located in Dortmund, Germany, where it was initiated at the Research Institute of Child Nutrition. Since 2012 the DONALD study has been carried out by the University of Bonn. Annual examinations include 3-d weighed dietary records, anthropometric measurements, collection of 24-h urine samples, interviews on lifestyle and medical examinations. This study was conducted according to the guidelines laid down in the Declaration of Helsinki and all procedures involving human subjects were approved by the Ethics Committee of the University of Bonn (ethics number: 098/06). Written informed consent was obtained from all parents and, later on, children. Details on the DONALD study design have been published previously^(^^14^^)^.

The sample size for the present evaluation was determined by all available complete dietary records from 3- to 18-year-old (>2·5–<18·5 years) participants, collected between 1985 and 2014. Hence, a total of 9757 dietary records from 1246 participants (629 boys and 617 girls) were included in the analytical sample. A total of 2620 dietary records (26·9 %) were available for the period 1985–1995, 3879 (39·7 %) for 1996–2005, and 3258 (33·4 %) for 2006–2014. Per participant, between one (*n* 156, 12·5 %) and sixteen (*n* 129, 10·4 %) dietary records were available.

### Dietary assessment

All foods and beverages consumed as well as leftovers were weighed and recorded over three consecutive days by the parents or by the older participants themselves with the use of electronic food scales. When exact weighing was not possible, household measures were allowed for semi-quantitative recording. Additionally, participants recorded the time of every eating occasion. Energy and protein intakes were calculated using our continuously updated in-house nutrient database LEBTAB.

Additionally, all foods and beverages consumed on the 3 d of recording were assigned to the following food groups: ‘starchy foods’ (bread, cereals, pasta, rice, potatoes), ‘fruits and vegetables’ (fresh/frozen/tinned products, juices), ‘dairy’ (milk, yoghurt, cheese), ‘meat, fish and eggs’ (fresh/frozen/tinned products), ‘fats & oils’ (spreads, plant oils), ‘legumes’, ‘nuts’, ‘non-core foods’ (sweets, cakes, savoury snacks), ‘vegetarian and vegan meat and dairy substitutes’ and ‘miscellaneous’ (beverages, seasoning). To this end, convenience food products (for example, canned soups, frozen pizza) were broken down into ingredients.

Morning intake was defined as all dietary intakes between an age-specific end of the night and 11.00 hours. Evening intake was defined as all dietary intakes between 18.00 hours and an age-specific beginning of the night^(^^10^^)^. Morning and evening protein intakes were considered as percentage of morning and evening energy intake (%E), respectively. Protein intakes from food groups in the morning and in the evening were considered as percentage of morning and evening protein intake, respectively. Subsequently, the individual means of protein and protein from food groups were calculated from the three record days.

### Assessment of potentially confounding factors

Body height (to the nearest 0·1 cm) and weight (to the nearest 100 g) were measured according to standard procedures with the participants dressed in underwear only and barefoot. BMI was calculated as the body weight (kg) divided by the square of the body height (m). Overweight, adiposity and underweight were defined according to International Obesity Task Force BMI cut-off-values for children and adolescents^(^^15^^,^^16^^)^.

Maternal body weight and height were also measured. Maternal overweight was defined as a BMI ≥25 kg/m^2^. High maternal educational status (≥12 years of schooling, general qualification for university entrance) and maternal employment (yes/no) were inquired with a standardised questionnaire. For missing values, the respective median of the total sample was used (*n* 31 for maternal overweight, *n* 5 for high maternal educational status, *n* 5 for maternal employment).

### Statistical analysis

All statistical analyses were performed using SAS procedures (version 9.2; SAS Institute, Inc.). The significance level was set at a *P* value of <0·05.

Time and age trends were investigated using polynomial mixed-effects regression models including both fixed and random effects (PROC MIXED in SAS^®^). Protein intake and protein from food groups in the morning and in the evening were considered in separate models as outcome variables. In cases of significant sex interactions, stratified analyses were performed. Age (continuously in years) and time (continuously in years; the first included record in this evaluation was considered the baseline time, i.e. time = 0) were the principal fixed effects. Quadratic and cubic terms for time (time^2^, time^3^) and age (age^2^, age^3^) as well as a combination of the linear age and time variable (age × time) were considered as additional explanatory variables, if they improved the fit statistics (Akaike information criterion) by more than two points or significantly predicted the respective outcome^(^^13^^)^. To account for the lack of independence between repeated measures from the same participant a repeated statement was considered. Additionally, random effects were considered to allow variation between individuals and families with respect to the initial level (intercept) as well as linear, quadratic and cubic age trends of the respective outcome. Subsequently, models were adjusted for the following potentially confounding factors: number of days with no dietary intake in the morning/evening per record (0/1/2/3), number of weekdays per record (1/2/3), participants’ body weight status (under-/normal-/overweight/adiposity), maternal overweight (BMI ≥25 kg/m^2^, yes/no), high maternal educational status (≥12 years of schooling, yes/no) and maternal employment (yes/no). To consider adequacy of recorded energy intake, the ratio between the recorded total daily energy intake (TEI) and the estimated BMR (according to the equations of Schofield^(^^17^^)^) (TEI:BMR) was calculated and considered as a potentially confounding factor. Variables that (1) significantly modified regression coefficients in the basic models by ≥10 %, (2) had a significant, independent effect on the outcome variable, or (3) led to an improvement of the Akaike information criterion by more than two points were considered in the final models.

The number of smoking adults per household – another potential confounder – was only available for 7703 measurements. To corroborate our results, we conducted sensitivity analyses, restricted to participants for whom data on the number of smokers in the household were available, adjusting for that potential confounder.

As single effect estimates of polynomial models cannot be interpreted, figures show the predicted outcome variables resulting from the polynomial mixed-effects regression models over the course of the study period for different ages.

## Results and discussion

To the best of our knowledge, this is the first study that investigated time and age trends in daytime-specific protein intakes in healthy children and adolescents. Characteristics of the study population are presented in Table 1. Evening protein intake was higher than morning protein intake among all age groups (Table 1). The major food groups contributing to protein intake in the morning and in the evening were ‘dairy’ (38 %/26 % of protein intake in the morning/evening), ‘starchy foods’ (36 %/28 % in the morning/evening) and ‘meat, fish & eggs’ (12 %/27 % in the morning/evening). While intakes of ‘dairy’ and ‘starchy’ protein were higher in the morning, intake of ‘meat, fish & eggs’ protein was higher in the evening (Table 1). Total daily protein intake ranged between 12·6 %/12·8 % of daily energy intake among 3- to 5-year-old boys/girls (i.e. 2·2/2·1 g/kg body weight) and 13·5 %/13·3 % among 14- to 18-year-old boys/girls (i.e. 1·2/1·0 g/kg body weight) (Table 1). The German recommended dietary allowance to meet the estimated average requirement of total protein intakes are age-specific (i.e. 1–3 years: 1·0 g/kg body weight; boys, 4–19 years and girls 4–14 years: 0·9 g/kg body weight; girls, 15–19 years: 0·8 g/kg body weight^(^^18^^)^).

**Table 1.** Sample characteristics of 9757 dietary records of 1246 Dortmund Nutritional and Anthropometric Longitudinally Designed (DONALD) study participants (3–18 years) between 1985 and 2014, stratified by sex (*n* 629 boys, *n* 617 girls) and age group (Numbers, medians with quartile 1 and quartile 3, or numbers and percentages)

	Boys								Girls							
	3–5 years		6–10 years		11–13 years		14–18 years		3–5 years		6–10 years		11–13 years		14–18 years	
	Median	Q1, Q3	Median	Q1, Q3	Median	Q1, Q3	Median	Q1, Q3	Median	Q1, Q3	Median	Q1, Q3	Median	Q1, Q3	Median	Q1, Q3
Diaries (*n*)	1419		1766		808		954		1381		1711		798		920	
Participants (*n*)	530		465		326		265		526		461		322		257	
Age (years)	4·0	3·1, 5·0	8·1	7·0, 9·2	12·1	11·3, 13·0	16·0	15·0, 17·1	4·1	3·1, 5·0	8·1	7·0, 9·1	12·1	11·2, 13·0	16·0	15·0, 17·1
Anthropometrics																
BMI (kg/m^2^)	15·6	14·9, 16·5	16·0	15·1, 17·5	18·4	16·7, 20·4	21·0	19·0, 23·2	15·5	14·7, 16·4	16·0	14·9, 17·6	18·2	16·6, 20·6	21·1	19·1, 23·1
Body weight status*																
Underweight																
*n*	184		135		62		62		167		396		83		92	
%	13·0		7·6		7·7		6·5		12		23·1		10·4		10	
Overweight																
*n*	82		180		122		153		121		224		99		110	
%	5·8		10·2		15·1		16·0		8·8		13·1		12·4		12	
Adiposity																
*n*	11		43		17		41		13		22		11		24	
%	0·8		2·4		2·1		4·3		0·9		1·3		1·4		2·6	
Dietary variables																
Total energy intake																
kJ/d	5217	4573, 5891	7037	6159, 7920	8284	7184, 9477	10025	8623, 11636	4766	4201, 5364	6293	5498, 7079	7376	6368, 8326	7360	6205, 8540
kcal/d	1247	1093, 1408	1682	1472, 1893	1980	1717, 2265	2396	2061, 2781	1139	1004, 1282	1504	1314, 1692	1763	1522, 1990	1759	1483, 2041
TEI:BMR	1·38	1·23, 1·53	1·47	1·31, 1·62	1·36	1·16, 1·53	1·32	1·12, 1·51	1·36	1·22, 1·51	1·42	1·26, 1·58	1·34	1·16, 1·52	1·19	1·01, 1·40
Daily protein intake																
%TEI	12·6	11·4, 13·9	12·8	11·5, 14·1	13·2	11·8, 14·6	13·5	12·1, 15·0	12·8	11·4, 14·2	12·5	11·2 13·9	12·9	11·5, 14·5	13·3	11·7, 15·0
g/kg body weight	2·2	1·9, 2·6	1·9	1·6, 2·2	1·4	1·2, 1·7	1·2	1·0, 1·4	2·1	1·8, 2·4	1·7	1·4, 1·9	1·3	1·0, 1·5	1·0	0·8, 1·2
Daily fat intake (%TEI)	35·6	31·6, 39·3	35·4	32·0, 39·0	35·4	31·6, 39·0	34·9	30·8, 39·2	35·7	32·4, 39·9	35·5	32·1, 39·0	35·3	32·1, 39·3	34·8	30·6, 38·5
Daily carbohydrate intake (%TEI)	51·6	47·5, 56·0	51·6	47·7, 55·5	51·3	47·5, 55·0	50·6	46·1, 55·4	51·0	47·0, 55·0	51·7	48·0, 55·7	51·6	47·3, 55·5	51·6	47·4, 55·9
Morning intake																
Energy intake (%TEI)	29·7	24·9, 35·2	27·8	23·0, 33·3	26·3	20·5, 32·1	23·6	16·6, 30·2	29·8	24·6, 35·5	28·2	23·4, 33·9	27·1	20·8, 32·4	25·0	18·1, 31·4
Protein intake (%E)†	12·6	10·7, 14·5	12·4	10·5, 14·2	12·3	10·4, 14·1	12·1	10·0, 14·2	12·7	10·9, 14·8	12·2	10·3, 14·1	12·2	10·1, 14·3	12·6	10·3, 14·8
‘Dairy’ protein (%)‡	45·0	28·7, 57·7	43·5	25·9, 58·0	36·5	20·3, 50·9	32·6	13·4, 48·2	43·6	27·8, 58·8	39·0	22·5, 53·2	33·7	16·2, 47·9	30·5	14·8, 45·5
‘Starchy foods’ protein (%)‡	31·1	21·7, 43·6	33·5	23·9, 45·1	36·1	26·2, 47·3	33·8	21·0, 45·9	31·1	21·1, 43·3	35·3	25·0, 47·1	37·4	26·5, 48·3	36·4	25·8, 47·8
‘Meat, fish & eggs’ protein (%)‡	8·4	0·0, 19·6	7·2	0·0, 18·0	8·9	0·0, 19·7	8·1	0·0, 17·8	9·3	0·0, 20·2	9·5	0·0, 20·3	10·2	0·0, 20·0	7·3	0·0, 18·8
Evening intake																
Energy intake (%TEI)	23·6	18·5, 28·6	27·2	22·0, 32·6	30·6	24·7, 36·4	32·8	26·7, 36·4	22·9	17·6, 28·2	25·6	20·0, 30·9	28·3	22·7, 34·3	29·0	22·3, 36·1
Protein intake (%E)†	14·0	12·0, 16·4	14·0	11·8, 16·3	13·9	11·8, 16·3	13·9	11·5, 16·4	14·4	12·2, 16·8	13·7	11·5, 15·8	13·6	11·2, 15·9	13·3	10·7, 16·1
‘Dairy’ protein (%)‡	27·1	13·1, 42·9	24·1	11·2, 39·3	24·4	12·7, 38·1	21·3	10·4, 34·4	25·3	10·8, 44·0	22·4	10·6, 37·6	21·9	10·5, 36·0	20·6	8·6, 35·4
‘Starchy foods’ protein (%)‡	25·8	16·8, 36·8	28·5	19·6, 39·3	24·7	17·6, 35·0	26·3	17·4, 34·6	24·3	15·3, 35·0	27·5	19·2, 37·5	26·5	18·5, 35·4	23·4	15·1, 33·3
‘Meat, fish & eggs’ protein (%)‡	24·6	10·2, 38·8	27·2	13·4, 40·3	27·8	15·1, 41·4	29·3	15·5, 43·0	23·5	11·4, 38·2	25·1	13·5, 39·8	26·0	13·2, 40·8	21·4	6·4, 37·7
Parental characteristics																
Maternal overweight§																
*n*	419		592		300		403		423		598		306		364	
%	29·5		33·5		37·1		42·2		30·6		35·0		38·4		39·6	
High maternal educational statusǁ																
*n*	969		1104		449		525		911		1039		450		504	
%	68·3		62·4		55·6		55·0		66·0		60·7		56·4		54·8	
Maternal employment																
*n*	588		1004		522		643		547		962		508		581	
%	41·4		56·9		64·6		67·4		39·6		56·2		63·7		63·2	

TEI, total energy intake; %TEI, percentage of total energy intake; %E, percentage of morning/evening energy intake.

* BMI cut off-values for children and adolescents for overweight and adiposity^(^^15^^)^ and underweight^(^^16^^)^.

† Percentage of morning/evening energy intake.

‡ Percentage of morning/evening protein intake.

§ BMI >25 kg/m^2^.

ǁ ≥12 Years of schooling.

**Fig. 1.**
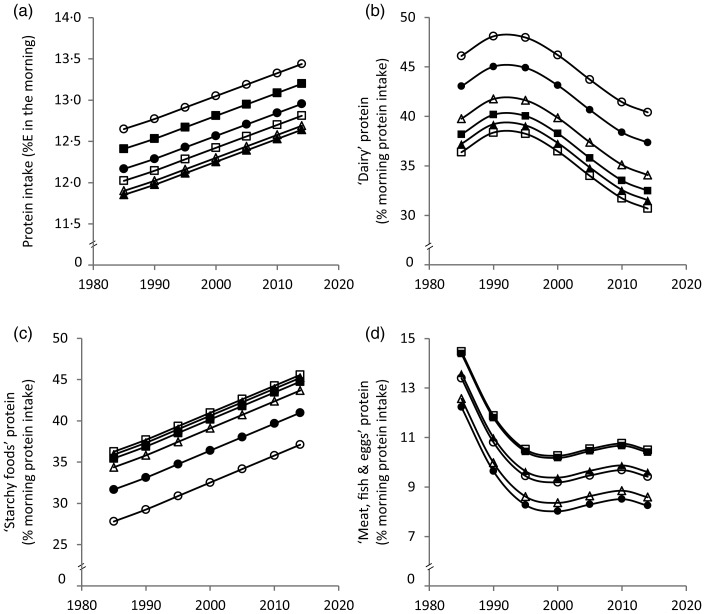
Time and age trends in morning (a) protein intake, (b) ‘dairy’ protein, (c) ‘starchy foods’ protein and (d) ‘meat, fish & eggs’ protein predicted from 9757 dietary records of 629 male and 617 female Dortmund Nutritional and Anthropometric Longitudinally Designed (DONALD) study participants (3–18 years) between 1985 and 2014, by use of polynomial mixed-effects regression models (see Table 2). ○, 3-year-olds, ●, 6-year-olds, Δ, 9-year-olds, ▲, 12-year-olds, □, 15-year-olds, ■, 18-year-olds. %E, percentage of morning energy intake.

**Fig. 2.**
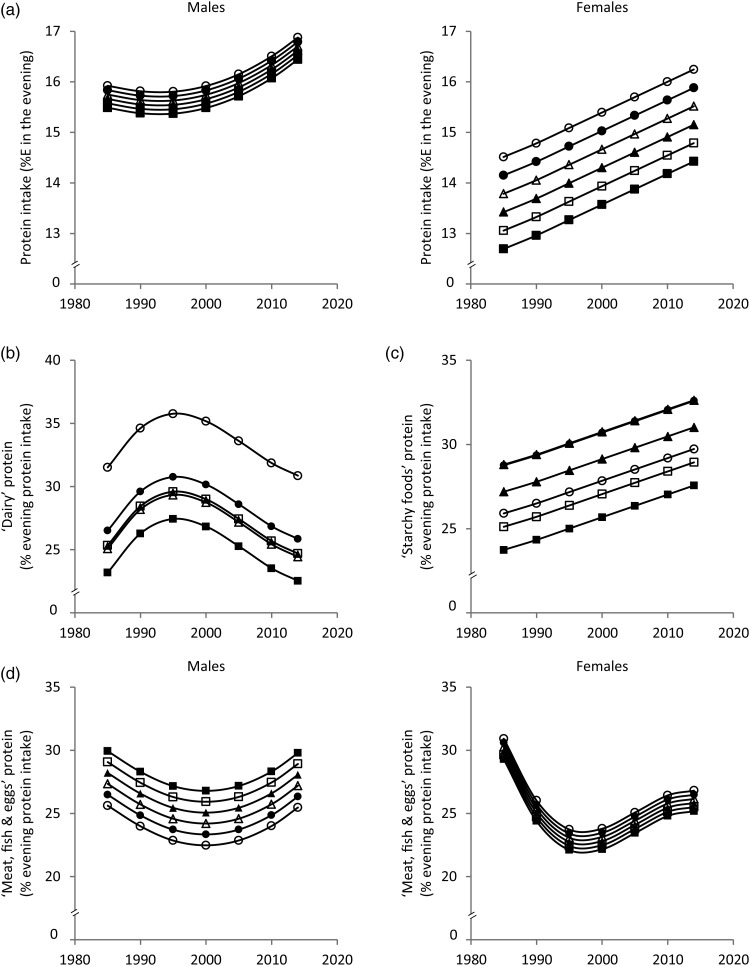
Time and age trends in evening (a) protein intake, (b) ‘dairy’ protein, (c) ‘starchy foods’ protein and (d) ‘meat, fish & eggs’ protein predicted from 9757 dietary records of 629 male and 617 female Dortmund Nutritional and Anthropometric Longitudinally Designed (DONALD) study participants (3–18 years) between 1985 and 2014, by use of polynomial mixed-effects regression models (see Table 2) ○, 3-year-olds, ●, 6-year-olds, Δ, 9-year-olds, ▲, 12-year-olds, □, 15-year-olds, ■, 18-year-olds. In cases of significant sex interactions, stratified analyses were performed. %E, percentage of evening energy intake.

Results from the polynomial mixed-effects regression models are shown in Table 2 and displayed in Figs 1 and 2. Morning protein intake increased between 1985 and 2014 by approximately 1%E among all age groups (Fig. 1(a)). The contribution of ‘dairy’ protein to morning protein increased slightly from 1985 to 1990 and decreased afterwards by approximately 8 % (Fig. 1(b)). The contribution of ‘starchy foods’ protein to morning protein intake in turn increased over time by approximately 10 % (Fig. 1(c)). The contribution of ‘meat, fish and eggs’ protein to morning protein decreased until the late 1990s by approximately 4 % and remained fairly stable thereafter (Fig. 1(d)).

**Table 2.** Time and age trends in morning and evening protein intakes, ‘dairy’ protein, ‘starchy foods’ protein and ‘meat, fish & eggs’ protein predicted from 9757 dietary records of 1246 Dortmund Nutritional and Anthropometric Longitudinally Designed (DONALD) study participants (*n* 629 boys, *n* 617 girls) (3–18 years) between 1985 and 2014 (β Regression coefficients with their standard errors†)

	Time trend per study year (1985–2014)						Age trend per year of age (3–18 years)					
	Time		Time × time		Time × time × time		Age		Age × age		Age × age × age	
	β	se	β	se	β	se	β	se	β	se	β	se
Morning intake												
Protein intake (% morning energy intake)‡	0·0278***	0·0061					−0·2699***	0·0361	0·0121***	0·0018		
‘Dairy’ protein (% morning protein intake)§	0·7241*	0·3306	−0·0676**	0·0245	0·0012*	0·0005	−0·3270	0·6976	−0·1183	0·0734	0·0059*	0·0024
‘Starchy foods’ protein (% morning protein intake)ǁ	0·3275***	0·0386					1·8754***	0·1989	−0·0650***	0·0096		
‘Meat, fish & eggs’ protein (% morning protein intake)¶	−0·7674**	0·2000	0·0442**	0·0148	−0·0008*	0·0003	−1·6502**	0·4325	0·1757**	0·0461	−0·0051**	0·0015
Evening intake												
Protein intake (% evening energy intake)												
Boys††	−0·0351	0·0363	0·0024*	0·0011			−0·0290	0·0174				
Girls‡‡	0·0609***	0·0100					−0·1214***	0·0171				
‘Dairy’ protein (% evening protein intake)§§	0·9657**	0·3035	−0·0645**	0·0225	0·0010*	0·0005	−4·6331***	0·6880	0·4122***	0·0729	−0·0118***	0·0023
‘Starchy foods’ protein (% evening protein intake)ǁǁ	0·1346***	0·0298					3·0828***	0·4994	−0·2875***	0·0533	0·0073***	0·0017
‘Meat, fish & eggs’ protein (% evening protein intake)												
Boys¶¶	−0·4365*	0·1823	0·0152**	0·0056			0·2872**	0·0902				
Girls†††	−1·4765**	0·3996	0·0901**	0·0299	−0·0015*	0·0007	−0·1084	0·0859				

* *P* < 0·05, ** *P* < 0·01, *** *P* < 0·0001.

† Regression coefficients with their standard errors result from polynomial mixed-effects regression models analysing linear, quadratic (time × time) and cubic (time × time × time) time trends as well as linear, quadratic (age × age) and cubic (age × age × age) age trends. There were no interactions between age and time (age × time) in all models.

‡ Adjusted for number of days with no dietary intake in the morning per record (0/1/2/3), ratio between total daily energy intake and estimated BMR, body weight status (under-/normal-/overweight/adiposity) and number of weekdays per record (1/2/3).

§ Adjusted for number of days with no dietary intake in the morning per record (0/1/2/3), number of weekdays per record (1/2/3) and ratio between total daily energy intake and estimated BMR.

ǁ Adjusted for number of days with no dietary intake in the morning per record (0/1/2/3), high maternal educational status (yes/no) and maternal overweight (yes/no).

¶ Adjusted for number of days with no dietary intake in the morning per record (0/1/2/3), number of weekdays per record (1/2/3), maternal overweight (yes/no) and body weight status (under-/normal-/overweight/adiposity).

†† Adjusted for number of days with no dietary intake in the evening per record (0/1/2/3), ratio between total daily energy intake and estimated BMR, number of weekdays per record (1/2/3), high maternal educational status (yes/no) and body weight status (under-/normal-/overweight/adiposity).

‡‡ Adjusted for number of days with no dietary intake in the evening per record (0/1/2/3), ratio between total daily energy intake and estimated BMR, and body weight status (under-/normal-/overweight/adiposity).

§§ Adjusted for number of days with no dietary intake in the evening per record (0/1/2/3), number of weekdays per record (1/2/3), ratio between total daily energy intake and estimated BMR, maternal overweight (yes/no) and high maternal educational status (yes/no).

ǁǁ Adjusted for number of days with no dietary intake in the evening per record (0/1/2/3), number of weekdays per record (1/2/3) and maternal employment (yes/no).

¶¶ Adjusted for number of days with no dietary intake in the evening per record (0/1/2/3), number of weekdays per record (1/2/3), high maternal educational status (yes/no) and maternal overweight (yes/no).

††† Adjusted for number of days with no dietary intake in the evening per record (0/1/2/3), ratio between total daily energy intake and estimated BMR, number of weekdays per record (1/2/3) and high maternal educational status (yes/no).

Evening protein intake increased between 1985 and 2014 in boys and girls by approximately 1%E and 2%E, respectively (Fig. 2(a)). The contribution of ‘dairy’ protein to evening protein slightly increased until the mid-1990s and decreased by approximately 5 % afterwards (Fig. 2(b)). The contribution of ‘starchy foods’ protein to evening protein increased over time by approximately 5 % (Fig. 2(c)). Among boys, the contribution of ‘meat, fish & eggs’ protein to evening protein showed a U-shaped time trend with a slight decrease until the late 1990s and a subsequent increase (±3 %) (Fig. 2(d)). Among girls, the contribution of ‘meat, fish & eggs’ protein to evening protein decreased until the late 1990s by approximately 8 % and increased afterwards by approximately 4 % (Fig. 2(d)). Sensitivity analyses additionally adjusting for the number of smokers in the household (data only available for 7703 measurements) yielded similar results (see Supplementary material).

The overall secular increase in both morning and evening protein intake was hence mainly attributable to the increased contribution of protein from ‘starchy foods’ rather than from traditional sources (i.e. dairy, meat or eggs) implying a shift from animal to plant protein in the morning and evening. In contrast to morning and evening protein intake, previous analyses of the DONALD study showed that total daily protein intake remained stable from 1985 to 2000^(^^12^^)^ and increased only slightly between 2000 and 2010 (0·06–0·11 %E/year)^(^^13^^)^ among DONALD study participants. Also total daily dairy intake remained stable at least until 2000^(^^12^^)^, suggesting that both the increases in morning and evening protein intakes and the decreases in morning and evening ‘dairy’ protein are partly compensated throughout the rest of the day. In contrast, secular increases in the contribution of protein from ‘starchy foods’ and decreases in the contribution of protein from ‘meat, fish & eggs’ to morning and evening protein probably mirror trends in total daily intakes of these food groups^(^^12^^)^. In the USA, daily %E from protein increased between 1973 and 1994 in 10-year-old children within the Bogalusa Heart Study^(^^19^^)^. However, at breakfast and dinner, no time trends were observed in bread and grain intake, inconsistent trends in beef, poultry, pork, mixed meats and eggs as well as no trends for milk, but increasing cheese intake^(^^20^^)^.

In terms of age trends, the youngest and the oldest children had the highest morning protein intake (Fig. 1(a)). While the contribution of ‘dairy’ protein to morning protein intake decreased from childhood to adolescence, the contribution of ‘starchy foods’ protein increased (Fig. 1(b) and (c)). In the evening, protein intake decreased with age among girls only (Fig. 2(a)). Similar to morning intake, the contribution of ‘dairy’ protein to evening protein decreased with age in the total sample (Fig. 2(b)). The contribution of ‘meat, fish & eggs’ protein to evening protein increased with age among boys only (Fig. 2(d)). The observed decrease in morning and evening ‘dairy’ protein and the increase in boys’ evening ‘meat, fish & eggs’ protein with age might in part reflect the transition from visiting kindergarten or primary school in childhood – a period characterised by consumption of many meals at home – to attending secondary school in adolescence accompanied by an increased consumption of food away from home. Furthermore, the growing autonomy during adolescence – also with respect to food choices – could be another factor.

Similar meal patterns at breakfast and dinner in Germany might explain similar time and age trends for morning and evening dietary intake. These trends suggest a replacement of ‘dairy’ by ‘starchy foods’ over time as well as with age. The fact that bread is generally the main component of breakfast and dinner in Germany might explain why ‘starchy foods’ emerged as a major source of morning and evening protein intake. In this study bread contributed 29·5 % of morning and 20·4 % of evening protein intake. The secular increase in protein from ‘starchy foods’ probably reflects the fact that the overall consumption of ‘starchy foods’ in the morning and in the evening increased over time (data not shown).

While a carbohydrate-rich diet is presently in line with recommendations, the rather low protein to carbohydrates and fat ratio of ‘starchy foods’ might not be favourable according to the protein leverage hypothesis^(^^1^^)^. Moreover, secular decreases in the quality of carbohydrates consumed by German adolescents, as reflected by decreases in daily fibre intake as well as increases in added sugars intake and glycaemic load^(^^21^^)^, might increase the risk for developing obesity and type 2 diabetes^(^^22^^)^. However, protein from plant sources has been suggested as protective against CVD as compared with protein from animal sources^(^^23^^)^.

Although daily dairy intake remained stable until 2000, its intake should be further monitored, since breakfast and dinner are the main occasions of dairy intake in Germany. Beside its relevance for growth, development and a healthy body weight status^(^^24^^)^ in children, a growing body of evidence suggests protection against the development of type 2 diabetes and CVD^(^^25^^)^. In this context, milk protein is discussed as one component having beneficial effects on metabolic health^(^^26^^)^.

The major limitation of the present study is the overrepresentation of families with a high socio-economic background, which limits the generalisability of our results. However, dietary patterns of DONALD study participants are similar to those of children and adolescents from a German representative survey^(^^13^^)^. Due to our relatively homogeneous sample, our results may be less vulnerable to residual confounding; however, residual confounding via unmeasured covariates remains a possibility. The strengths of this study include the longitudinal design with a long follow-up, the repeated measurements on the same individual and the detailed assessment of the dietary intake. However, there might be differences in dietary data recorded by parents or participants themselves. While children are suggested to reliably report their dietary intake from the age of 8–10 years, parents reliably report food intake at home, but often do not know about consumption outside of the home^(^^27^^)^. Additionally, due to the fact that only morning and evening intakes were considered in this analysis, conclusions on the compensation of food intake at other day-times can only be drawn cautiously when comparing with studies on trends in daily intakes. The comparability with these studies is, however, limited.

In conclusion, morning and evening protein intakes increased modestly between 1985 and 2014. Interestingly, these increases were not accompanied by secular increases in the contribution of protein from traditional sources to morning and evening protein intakes, instead protein from ‘starchy foods’ became a more relevant protein source. In particular, decreases in the relative contribution of morning and evening ‘dairy’ protein to total protein require further monitoring.

## Supplementary material

For supplementary material accompanying this paper visit https://doi.org/10.1017/jns.2018.1 click here to view supplementary material
