# Supplementary material for: Time and age trends in morning and evening protein intakes of German children and adolescents
Source: J Nutr Sci. 2018 Mar 22;7:e9. doi: 10.1017/jns.2018.1 (PMC5869278; doi:10.1017/jns.2018.1)
Supplement: Supplementary file 1 [file S2048679018000010sup001.docx]

**Supplementary material**

**Time and age trends in morning and evening protein intakes of German children and adolescents**

*Journal of Nutritional Science*

Sarah Roßbach, Tanja Diederichs, Christian Herder, Anette E. Buyken, Ute Alexy^*^

*Corresponding author; IEL-Nutritional Epidemiology, DONALD Study, University of Bonn, Heinstueck 11, 44225 Dortmund, Germany; E-mail address: alexy@uni-bonn.de

**Supplementary Table S1.** Time and age trends in morning and evening protein intake, ‘dairy’ protein, ‘starchy foods’ protein and ‘meat, fish & eggs’ protein predicted from 7,703 dietary records of 523 male and 490 female DONALD study participants (3-18 years) between 1985 and 2014 with additional adjustment for the number of smoking adults in the household^a^

|  | **Time trend per study year**  (1985-2014) | | | | | | **Age trend per year of age**  (3-18 years) | | | | | |
| --- | --- | --- | --- | --- | --- | --- | --- | --- | --- | --- | --- | --- |
|  | Time | | Time*Time | | Time*Time*Time | | Age | | Age*Age | | Age*Age*Age | |
|  | β | SE | β | SE | β | SE | β | SE | β | SE | β | SE |
| *Morning intake* |  |  |  |  |  |  |  |  |  |  |  |  |
| Protein intake (%E) ^b, c^ | 0.0255† | 0.0068 |  |  |  |  | -0.2388‡ | 0.0395 | 0.0102‡ | 0.0020 |  |  |
| ‘Dairy’ protein (%) ^d, e^ | 0.5912 | 0.3678 | -0.0583* | 0.0270 | 0.0011 | 0.0006 | -0.1833 | 0.7791 | -0.1263 | 0.0821 | 0.0059* | 0.0027 |
| ‘Starchy foods’ protein (%) ^d, f^ | 0.3344‡ | 0.0410 |  |  |  |  | 1.8671‡ | 0.2179 | -0.0612‡ | 0.0105 |  |  |
| ‘Meat, fish & eggs’ protein (%) ^d, g^ | -0.7160† | 0.2235 | 0.0431† | 0.0165 | -0.0008* | 0.0004 | -1.6399† | 0.4879 | 0.1700† | 0.0523 | -0.0049† | 0.0017 |
| *Evening intake* |  |  |  |  |  |  |  |  |  |  |  |  |
| Protein intake (%E) ^b^ |  |  |  |  |  |  |  |  |  |  |  |  |
| Boys ^h^ | -0.0148 | 0.0403 | 0.0019 | 0.0012 |  |  | -0.0396* | 0.0185 |  |  |  |  |
| Girls ^i^ | 0.0534‡ | 0.0115 |  |  |  |  | -0.1130‡ | 0.0196 |  |  |  |  |
| ‘Dairy’ protein (%) ^d, j^ | 0.9340† | 0.3425 | -0.0649* | 0.0254 | 0.0011 | 0.0005 | -5.6292‡ | 0.7697 | 0.5146‡ | 0.0816 | -0.0151‡ | 0.0026 |
| ‘Starchy foods’ protein (%) ^d, k^ | 0.1459‡ | 0.0321 |  |  |  |  | 3.3121‡ | 0.5599 | -0.3109‡ | 0.0600 | 0.0081‡ | 0.0019 |
| ‘Meat, fish & eggs’ protein (%) ^d^ |  |  |  |  |  |  |  |  |  |  |  |  |
| Boys ^l^ | -0.2421 | 0.2038 | 0.0093 | 0.0062 |  |  | 0.2629† | 0.0966 |  |  |  |  |
| Girls ^m^ | -1.7468† | 0.4577 | 0.1134† | 0.0343 | -0.0021† | 0.0007 | -0.1311 | 0.0946 |  |  |  |  |

β, regression coefficient; SE, standard error; %E, percentage of morning/evening energy intake.

^a^ Values presented are regression coefficients with standard errors resulting from polynomial mixed-effects regression models analyzing linear, quadratic (time*time) and cubic (time*time*time) time trends as well as linear, quadratic (age*age) and cubic (age*age*age) age trends.

^b^ Percentage of morning/evening energy intake.

^c^ Adjusted for number of days with no dietary intake in the morning per record (0/1/2/3), ratio between total daily energy intake and estimated basal metabolic rate, body weight status (under-/normal-/overweight/adiposity), number of weekdays per record (1/2/3), number of smoking adults in the household.

^d^ Percentage of morning/evening protein intake.

^e^ Adjusted for number of days with no dietary intake in the morning per record (0/1/2/3), number of weekdays per record (1/2/3), ratio between total daily energy intake and estimated basal metabolic rate, number of smoking adults in the household.

^f^ Adjusted for number of days with no dietary intake in the morning per record (0/1/2/3), high maternal educational status (yes/no), maternal overweight (yes/no), number of smoking adults in the household.

^g^ Adjusted for number of days with no dietary intake in the morning per record (0/1/2/3), number of weekdays per record (1/2/3), maternal overweight (yes/no), body weight status (under-/normal-/overweight/adiposity), number of smoking adults in the household.

^h^ Adjusted for number of days with no dietary intake in the evening per record (0/1/2/3), ratio between total daily energy intake and estimated basal metabolic rate, number of weekdays per record (1/2/3), high maternal educational status (yes/no), body weight status (under-/normal-/overweight/adiposity), number of smoking adults in the household. With the reduced sample size, the quadratic time trend was not relevant anymore. When excluding the quadratic trend, the linear trend was significant again (ß=0.04386, p=0.0005) confirming the secular increase in evening protein intake among boys.

^i^ Adjusted for number of days with no dietary intake in the evening per record (0/1/2/3), ratio between total daily energy intake and estimated basal metabolic rate, body weight status (under-/normal-/overweight/adiposity), number of smoking adults in the household.

^j^ Adjusted for number of days with no dietary intake in the evening per record (0/1/2/3), number of weekdays per record (1/2/3), ratio between total daily energy intake and estimated basal metabolic rate, maternal overweight (yes/no), high maternal educational status (yes/no), number of smoking adults in the household.

^k^ Adjusted for number of days with no dietary intake in the evening per record (0/1/2/3), number of weekdays per record (1/2/3), maternal employment (yes/no), number of smoking adults in the household.

^l^ Adjusted for number of days with no dietary intake in the evening per record (0/1/2/3), number of weekdays per record (1/2/3), high maternal educational status (yes/no), maternal overweight (yes/no), number of smoking adults in the household.

^m^ Adjusted for number of days with no dietary intake in the evening per record (0/1/2/3), ratio between total daily energy intake and estimated basal metabolic rate, number of weekdays per record (1/2/3), high maternal educational status (yes/no), number of smoking adults in the household.

* P<0.05

† P<0.01

‡ P<0.0001
